# Do girls wash dishes and boys play sports? Gender inequalities in physical activity and in the use of screen-based devices among schoolchildren from urban and rural areas in Brazil

**DOI:** 10.1186/s12889-024-17672-1

**Published:** 2024-01-16

**Authors:** Gilmar Mercês de Jesus, Lizziane Andrade Dias, Anna Karolina Cerqueira Barros, Lara Daniele Matos dos Santos Araujo, Mayva Mayana Ferreira Schrann

**Affiliations:** Public Health Post-Graduate Program, State University of Feira de Santana, Travessa Pássaro Vermelho,32, Santa Mônica II, CEP: 44082- 320 Feira de Santana, Brazil

**Keywords:** Physical activity, Gender equity, Children, Adolescents, Ethnicity

## Abstract

**Objective:**

The aim of the study was to analyze gender inequalities in types of physical activity (PA) and in the use of screen-based devices among schoolchildren from both urban and rural areas in Brazil.

**Methods:**

Data from two population-based surveys conducted in 2019 (urban areas: *n* = 2,479; 52.6% girls; age = 9.2 ± 1.51 years) and 2022 (rural areas: *n* = 979; 42.6% girls; age = 9.4 ± 1.52 years) were used. PA (active play, nonactive play, home chores, and structured physical activities) and the use of screen-based devices (TV, cellphone, videogame, and computer) were self-reported in a previous-day-recall online questionnaire (Web-CAAFE). Absolute gender inequalities were evaluated and presented as equiplots. Relative gender inequalities were evaluated by the prevalence ratio (PR) and respective 95% confidence intervals (95% CI), which were estimated by Poisson regression, with adjustments for age and BMI z scores.

**Results:**

Girls from urban and rural areas presented a lower prevalence of active play and a higher prevalence of home chores. The prevalence of nonactive play among girls from urban areas was also lower; however, their prevalence of structured physical activities was higher, especially among girls aged seven to nine years. Girls in both urban and rural areas presented a higher prevalence of TV viewing and lower use of video games.

**Conclusion:**

The gender inequalities observed in the types of physical activities and in the use of screen-based devices could be considered potential correlates of the likelihood of girls’ and boys’ compliance with the physical activity guidelines.

**Supplementary Information:**

The online version contains supplementary material available at 10.1186/s12889-024-17672-1.

## Background

Gender is a relevant determinant of health inequality, influencing behaviors, healthcare practices, and several health outcomes [1]. Generally, gender inequalities in health are a consequence of the additional suffering of women due to a set of disabling physical and mental illnesses, when compared to men, even though they have longer life expectancy [2].

Despite considerable advancements in civilization over recent decades, gender inequalities remain a pressing issue for the development of nations worldwide [3]. More recently, the COVID-19 pandemic has worsened these inequalities, leading to increased rates of unemployment, incidents of domestic and sexual violence [4] and even physical inactivity among women [5].

Physical inactivity is recognized as a global pandemic that contributes to 5.3 million deaths per year [6]. In Brazil, approximately 32,400 deaths from all cardiovascular causes have been attributed to physical inactivity, underscoring the imperative to invest in strategies and policies to promote physical activity within the Brazilian population to prevent early mortality [7].

The participation of men and women in physical activities is also unequal and the prevalence of adults [8] and adolescents [9, 10] who do not meet physical activity guidelines is higher among women and girls worldwide. Data from a pooled analysis 298 school-based surveys from 146 countries showed that 87.4% of girls aged 11–17 years were insufficiently physically active compared to 77.6% of boys.

In Brazil, Araújo and colleagues [11], analyzing data from 2006 to 2019 obtained from the Brazilian System for Surveillance of Risk and Protective Factors for Chronic Diseases via Telephone Survey (*Vigilância de Fatores de Risco e Proteção para Doenças Crônicas por Inquérito Telefônico– VIGITEL Brasil*), identified persistent gender inequalities in participation in different types of physical activity over leisure time. Their findings revealed a higher participation of women in activities such as walking and gym-based exercises but lower involvement in sports and other leisure-time physical activities.

Among adolescents participating in the National School Health Survey (*PeNSE– Pesquisa Nacional de Saúde do Escolar*), the percentage of girls meeting the recommendation of 60 min of moderate to vigorous physical activity during leisure time was also lower in all editions of the survey (2009, 2012 and 2015) [12]. PeNSE data revealed that from 2009 to 2012 the percentage of girls meeting physical activity guidelines was almost 2 times lower than boys, being almost three times lower in 2019, when the lowest percentages of physically active adolescents were recorded [12].​ However, the engagement of Brazilian adolescents in different types of physical activities and sedentary behaviors is still a gap in the PeNSE survey.

Physical activity is a complex behavior that varies across the population with respect to time, intensity, frequency, type, and context and shows evidence of tracking from childhood/adolescence [13] to adulthood [14]. This indicates that physically active children are more likely to remain that way into adulthood.

The range of correlates and determinants of sedentary behaviors, especially exposure to screen-based devices, is also complex, and there is evidence of tracking this behavior [13], similar to physical activity; therefore, its monitoring is important, given its association with unfavorable health outcomes. Therefore, the factors that restrict girls’ participation in physical activities during childhood and adolescence and those that favor exposure to screens may be the origin of women’s lower participation in physical activities during adulthood observed in population studies [9–11].

Cultural gender norms and pressures to appear feminine can decrease girls’ opportunities to engage in many types of physical activities and sports and may even explain why they are less physically active than boys. The available evidence shows girls’ higher involvement in sedentary behaviors [15], light physical activities [16], and rhythmic and expressive activities, however their participation in sports is lower than that of boys [17].

​In a previous study, we described gender differences in the types of physical and sedentary activities among children and adolescents, and we found that vigorous activities, such as soccer and wrestling, were more self-reported by boys, while dancing, ballet, jumping rope and household chores predominated among girls [18]. Although these findings highlight striking gender-related disparity issues in physical activities, our previous study carried out in 2019 is limited by its sample composition, which consists solely of students from a single public urban school, restricting its generalization.

Assessing the most common physical and sedentary activities among children provides strategic information. It allows us to know in a swift, practical, and objective means to understand the preferences and contexts of these behaviors. These aspects are influenced by culture, personal skills, and most significantly, by inequalities in access to suitable facilities and equipment for the practice [11].

In addition, to the best of our knowledge, it remains unknown whether gender inequalities in children’s and adolescents’ participation in types of physical activities and sports are similar when comparing schoolchildren from urban and rural areas in Brazil. Therefore, the present study aims to analyze gender inequalities in types of physical activity and in the usage of screen-based devices among schoolchildren from urban and rural areas in Brazil.

## Methods

### Design

This study employs a cross-sectional design using data gathered from two school-based surveys conducted in both the urban and rural areas of Feira de Santana, Bahia, in 2019 and 2022, respectively. Data collection took place from March to October 2019 and from July to December 2022 on weekdays (from Tuesday to Thursday) during school hours.

Feira de Santana is the most populous and most economically efficient city, located in the interior of the state of Bahia, which boasts a population of 616,279 inhabitants in 2022 (IBGE, 2022, available at: https://www.ibge.gov.br/cidades-e-estados/ba/feira-de-santana.html). In 2019, the Municipal Department of Education reported a total of 205 schools within the city’s municipal public school system: out of these, 163 offered classes from the 2nd to the 5th grade of elementary school, and 122 were situated in urban areas. Our survey targeted a sample of students from these schools. The calculated sample size needed 2,000 students, considering a population of 15,920 students enrolled from the 2nd to the 5th grade in the city’s public schools, expected prevalence of the outcome of 50%, margin of error set at 3% points and design effect of 2.0. Moreover, a 20% buffer was incorporated to account for potential losses and refusals, resulting in a targeted sample of 2,400 students.

Cluster sampling was carried out in three stages: (I) stratification of all municipal schools based on 11 geographic and administrative centers, which categorize schools by geographical position and guide municipal actions; (II) random selection of one school from each center; and (III) selection of participating classes (2nd to 5th grade) within each school, totaling 159 classes.

The survey carried out in 2022 included a sample of students hailing from rural areas within Quilombola communities. Feira de Santana comprised eight rural districts (*Ipuaçu*, *Bonfim de Feira*, *São José*, *Humildes*, *Tiquaruçu*, *Jaíba*, *Jaguara* e *Matinha*) and three Quilombola communities certified as per Presidential Decree No. 4887/2003: *Lagoa Grande* (São José District), *Matinha dos Pretos* and *Fazenda Candeal II* (Matinha District). According to the Municipal Department of Education data, in 2022, there were 19 Quilombola schools offering classes from the 2nd to the 5th grade, with 16 of them situated in rural areas. In these schools, 2,714 students were enrolled (2,138 in rural schools).

The sample size of participants from rural schools was calculated considering the following parameters: population of 2,138 students, expected outcome prevalence of 19.87% of estimated overweight among children and adolescents from Feira de Santana, margin of error set at 3% points, and a design effect of 1.5. Based on these parameters, the calculated sample size was 774 individuals. Moreover, a 20% adjustment was incorporated to account for potential losses and refusals, resulting in a final targeted sample of 929 students.

**Fig. 1:**
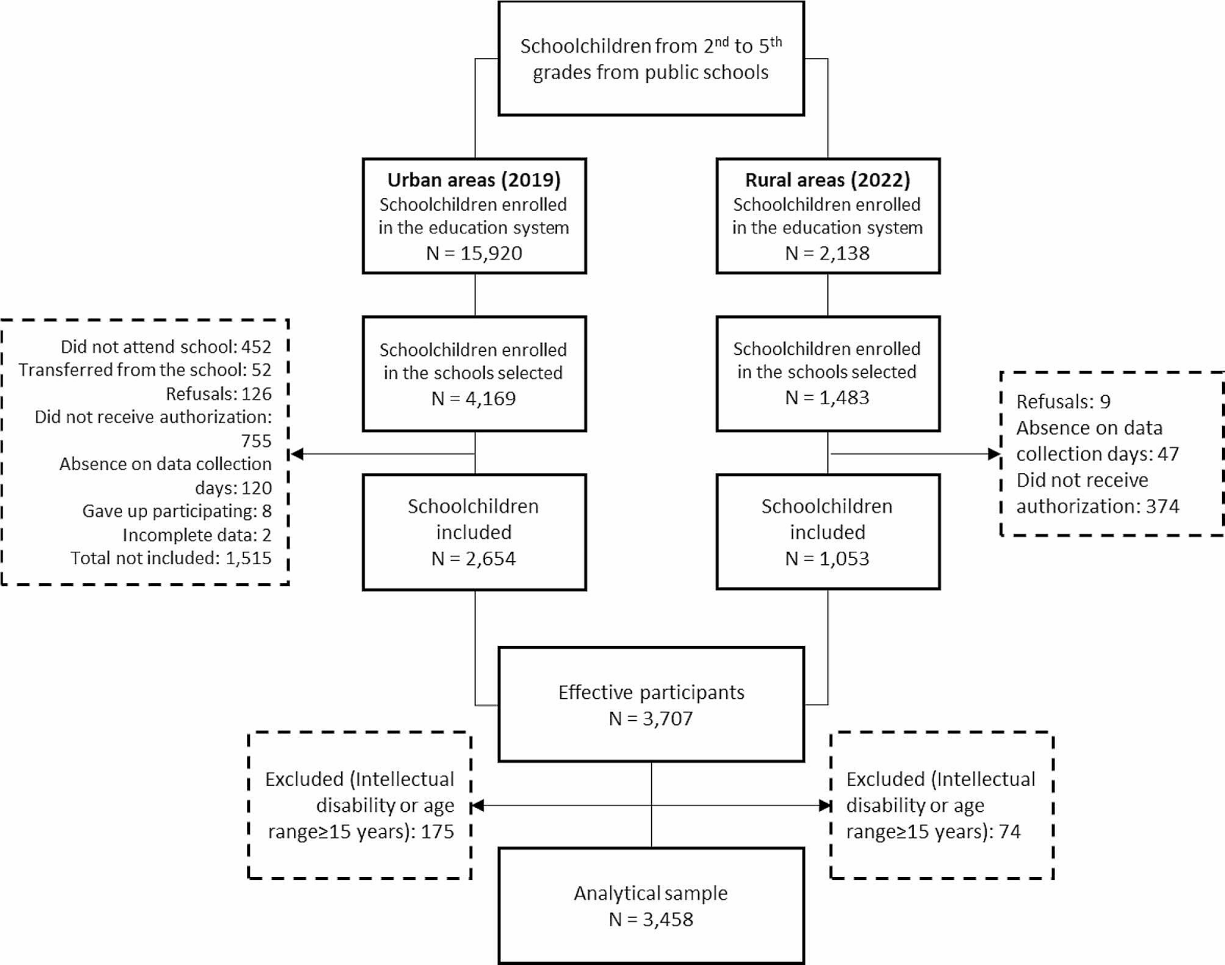
Study flowchart

In the 2022 survey, the cluster sampling process was carried out in four stages: (I) stratification of all rural Quilombola schools based on their respective districts; (II) computation of the total student count within each district (conglomerate) by summing the enrollments in the selected schools, with each school’s weight within the conglomerate calculated as a percentage; (III) determination of the desired sample size in each school by multiplying the calculated sample size by the corresponding percentage value reflecting the school’s weight within the cluster; and (IV) selection of participating classes (2nd to 5th grade) within each school, totaling 83 classes, and random drawing of the needed number of students. Figure 1 informs on the composition of the analytical sample according to inclusion and exclusion criteria.

### Participants

All students enrolled in the 2nd to 5th grade classes of the respective participating schools in 2019 and 2022 received invitations to participate. Inclusion criteria encompassed regular attendance, written parental authorization (mandatory for all participants under 16 years), and voluntary consent obtained through reading and signing the consent form. Both studies followed the ethical guidelines outlined in Brazilian National Health Council (*CNS - Conselho Nacional de Saúde*) Resolution No. 466/2012 and received approval from the Research Ethics Committee of the State University of Feira de Santana (2019: CAAE: 02307918.5.0000.0053/opinion number − 3.116.495; 2022: CAAE: 29137320.6.0000.0053/opinion number − 3.994.186). Children and adolescents with intellectual disabilities and those falling outside the age range between seven and 12 years old were included in the studies but excluded from the following statistical analyses.

### Measurements of physical activity and screen-based device usage

Physical activity and screen-based device use were assessed via an online self-report questionnaire, which relies on previous-day recall: Web-CAAFE (available at: https://caafe.ufsc.br/portal/9/detalhes). Web-CAAFE has been validated in two Brazilian cities [19–21] and provides information on various aspects, including food consumption, weight status, physical activities (including commuting to/from school), and screen-based device usage.

Overall, Web-CAAFE displays up to 32 items representing physical activities and sedentary behaviors, including the use of screen-based devices (TV, video games, computers, and cell phones). Each participant was asked to reportthe activities performed in three shifts of the previous day (morning, afternoon, and evening). Participants completed the Web-CAAFE at the school, in a room with laptops and headphones, after having been instructed about how the software works and how to complete the questionnaire.

Information regarding the gender and age of the participants was obtained from the Municipal Department of Education. In the present study, academic activities were not included in the analyses.

### Anthropometric measurements and overweight assessment

Both surveys, in 2019 and 2022, included body weight and height measurements to calculate body mass index (BMI), measured by trained researchers, following recommended standardization [22]. Weight was measured using a portable digital scale, brand AVAnutri®, with a precision of 100 g and a maximum capacity of 200 kg. Height was measured using a portable, detachable stadiometer from Seca® with a platform and square, offering a maximum height of 205 cm and precision of 1 mm. During the measurements, participants were barefoot, wearing the school uniform, and with no headwear. Sex- and age-specific BMI z score values were calculated according to International Obesity Task Force (IOTF) curves [23]. Weight status was classified into underweight, overweight, and obesity.

### Potential confounders

Engagement in different types of physical activities and the use of different electronic screen-based devices can be influenced by factors such as age range and overweight. Therefore, we included these variables in our adjusted modeling. Previous studies have demonstrated a decrease in physical activity levels from childhood to adolescence [24, 25]. This phenomenon may include a waning interest in certain types of physical activities. On the other hand, exposure to screen-based devices is often described as high among adolescents compared to children [26, 27].

Furthermore, although there is evidence of a bidirectional association between overweight and physical activity, it is relevant to consider findings indicating that being overweight may restrict the participation of children and adolescents in specific types of physical activity [28].

### Data processing and analysis

We conducted data analysis using Stata 15 software (Stata Corp 2017, College Sataion, TX, USA). Our analysis included descriptive statistics to determine frequencies with proportions and 95% confidence intervals (95%CI), as well as means with standard deviations (± SD). To assess inequalities, we calculated absolute differences (diff.) in percentage points (p.p.) for the proportions of different physical activity types and screen-based device usage. These differences were presented as absolute values and visualized using equiplots (https://equidade.org/equiplot).

Relative inequalities were assessed through prevalence ratios (PR) and 95% CI estimated by Poisson regression, adjusted for age and overweight (overweight + obesity). We considered a significance level of *p* < 0.05 as the threshold for determining statistical significance. Missing data were described as relative frequencies, and the subjects’ characteristics were compared with those who remained in the analytical sample by chi-square (χ²) statistics. For the data analysis, physical activities and screen-based device usage were classified into the following subgroups, which were formed by the sum of the daily reports of the respective items:

- *Active play* (playing with a ball, play catch-up, soccer, dance, marbles, jump rope, gymnastics, elastics, playing in the park, playing in the water/swimming, ride a bicycle, rollerblading/skateboarding/riding a scooter, flying a kite, dodgeball, hide-and-seek, playing with dog, and hopscotch).
- *Nonactive play* (board games, playing with dolls/action figures, playing with toy cars, spinning top/bayblade, listening to music, playing musical instrument).
- *Structured physical activities* (ballet, fighting sports),
- *Household chores* (washing dishes, sweeping).
- *Screens* (TV, cell phone, computer, and video game).

## Results

In this study, the analytical sample was composed of 3,458 students (Fig. 1) (2,479 from urban schools: age = 9.1 ± 1.38 years, 19.9% overweight, 53.2% girls; and 979 from rural schools: age = 9.3 ± 1.39 years; 24% overweight, 49.8% girls).

Table 1 describes the prevalence of physical activities and use of electronic screen-based devices among the participants. Cell phone and TV use were the items most reported by children in urban and rural areas, reaching a prevalence close to 50%. Then, the activities with the most reports were soccer, play catch-up, washing dishes and sweeping.

**Table 1:** Prevalence and 95% confidence intervals of physical activities and use of screen-based devices among children from urban and rural areas. Feira de Santana, Bahia, Brazil

Physical activities and screen-based devices	Urban areas (*n* = 2,479)		Rural areas (*n* = 979)	
	P (%)	95% CI	P (%)	95% CI
TV	46.7	44.7–48.6	41.1	38.0-44.2
Cell phone	49.0	47.0–51.0	44.3	41.2–47.5
Computer	11.5	10.3–12.8	5.9	4.6–7.6
Video game	9.7	8.2–10.9	4.9	3.7–6.6
Playing marbles	8.9	7.8–10.1	9.1	7.4–11.1
Board games	4.0	3.3–4.8	2.4	1.6–3.5
Playing with dolls/action figures	12.6	11.3–14.0	8.2	6.6–10.1
Playing with toy cars	4.6	3.8–5.4	5.0	4.0-6.8
Spinning top/bayblade	8.7	7.7–9.9	5.2	4.0-6.8
Listening to music	9.8	8.7–11.0	5.0	3.8–6.6
Playing musical instrument	3.1	2.5–3.9	1.7	1.1–2.8
Play catch-up	26.8	25.1–28.6	25.9	23.3–28.8
Dancing	7.9	6.9–9.1	4.4	3.3–5.9
Hopscotch	8.8	7.7–10.0	9.3	7.6–11.3
Gymnastics	7.5	6.5–8.6	4.9	3.7–6.4
Elastics	4.2	3.5–5.1	2.2	1.5–3.4
Playing in the park	7.7	6.7–8.8	4.1	3.0-5.5
Playing in the water/Swimming	7.9	6.9-9.0	3.7	2.7–5.1
Rollerblading/Skateboarding/Riding a scooter	6.2	5.3–7.2	1.7	1.1–2.8
Flying a kite	3.4	2.7–4.1	9.1	7.4–11.1
Dodgeball	3.9	3.2–4.8	3.2	2.2–4.5
Hide and seek	13.6	12.3–15.0	11.8	9.9–13.9
Playing with a dog	8.4	7.3–9.5	9.0	7.4–11.0
Sweeping	20.1	19.2–22.4	18.5	16.2–21.1
Washing dishes	21.1	19.5–22.7	20.9	18.5–23.6
Playing with a ball	5.0	4.2–5.9	4.1	3.0-5.5
Soccer	28.5	26.7–30.3	30.3	27.5–33.3
Ballet	7.7	6.7–8.8	2.4	1.6–3.6
Fighting sports	8.0	6.9–9.1	3.9	2.8–5.3
Jumping rope	13.0	11.8–14.4	8.9	7.3–10.8
Riding a bicycle	10.6	9.4–11.8	12.6	10.6–14.8

P: Prevalence. 95% CI: 95% confidence interval

### Gender inequalities in physical activity and use of screen-based devices in urban areas

Gender disparities were noted among urban schoolchildren in activities such as playing with a ball, sweeping, washing dishes, jumping rope, ballet, playing catch-up, and playing with dolls/action figures (Table 1S Supplementary Material A). These activities were predominantly reported by girls, with absolute differences exceeding 10 p.p. when compared to boys. Soccer (diff > 46 p.p.), spinning top/beyblade and video games were mostly self-reported by boys.

Gender differences in relative values can be found in Table 1S. There was no statistically significant gender difference for cell phone use, listening to music, playing in the water/swimming, rollerblading/skateboarding/riding a scooter, and playing with dog.

In urban areas, girls reported 11% less engagement in active play and 16% less involvement in nonactive play compared to boys. Conversely, the prevalence of household chores among girls was twice as high as that among boys. There was no statistically significant difference in the self-reports of *screens* (Fig. 3).

Age modified the effect of gender on structured physical activities. We found that girls aged 7–9 (PR = 1.51; 95% CI = 1.20–1.90) had a higher prevalence of engagement in these activities than girls aged 10–12. Additionally, girls exhibited a higher prevalence of structured physical activities than boys, regardless of age.

### Gender inequalities in physical activity and use of screen-based devices in rural areas

**Fig. 2:**
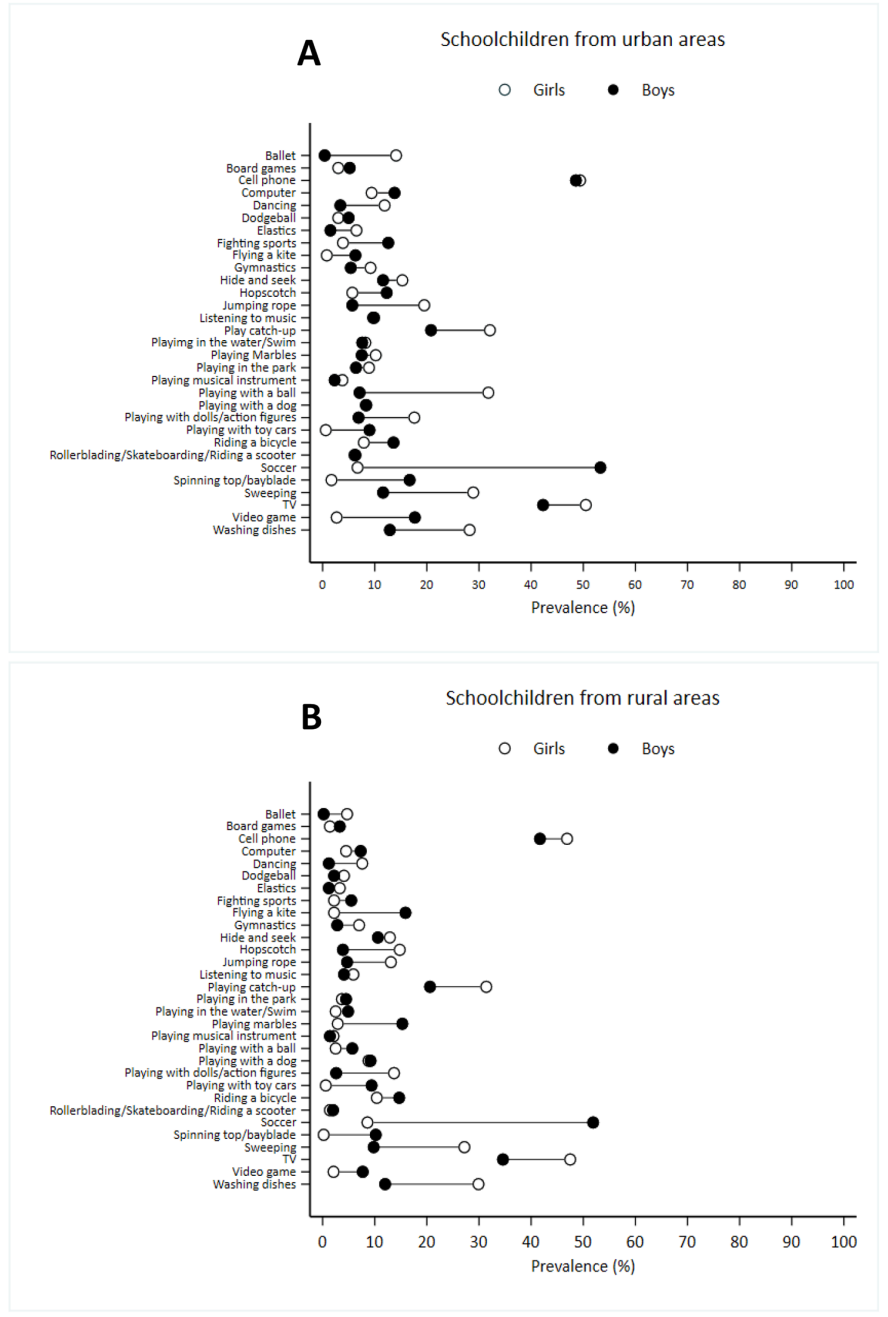
Absolute gender inequalities in physical activities and use of screen-based devices among schoolchildren from urban (A) and rural (B) areas. Feira de Santana, Bahia, Brazil

Among the schoolchildren in rural areas, girls self-reported higher engagement in activities such as washing dishes, sweeping, watching TV, playing with dolls/action figures, hopscotch and play catch-up, with prevalence more than 10 p.p. higher than those shown by boys for these items (Fig. 2). Additionally, boys in rural areas also self-reported a higher participation in activities such soccer, with prevalence > 43 p.p higher when compared to girls. Boys also self-reported higher frequency of activities such as flying a kite, playing marbles, and spinning top/Beyblade than girls. There was no statistically significant difference in the self-reports of cell phone, computer, board games, listening to music, playing musical instrument, playing in the park, rollerblading/skateboarding/riding a scooter, dodgeball, playing hide and seek, playing with a dog, and riding a bicycle (Table 2 S - Supplementary material B).

**Fig. 3:**
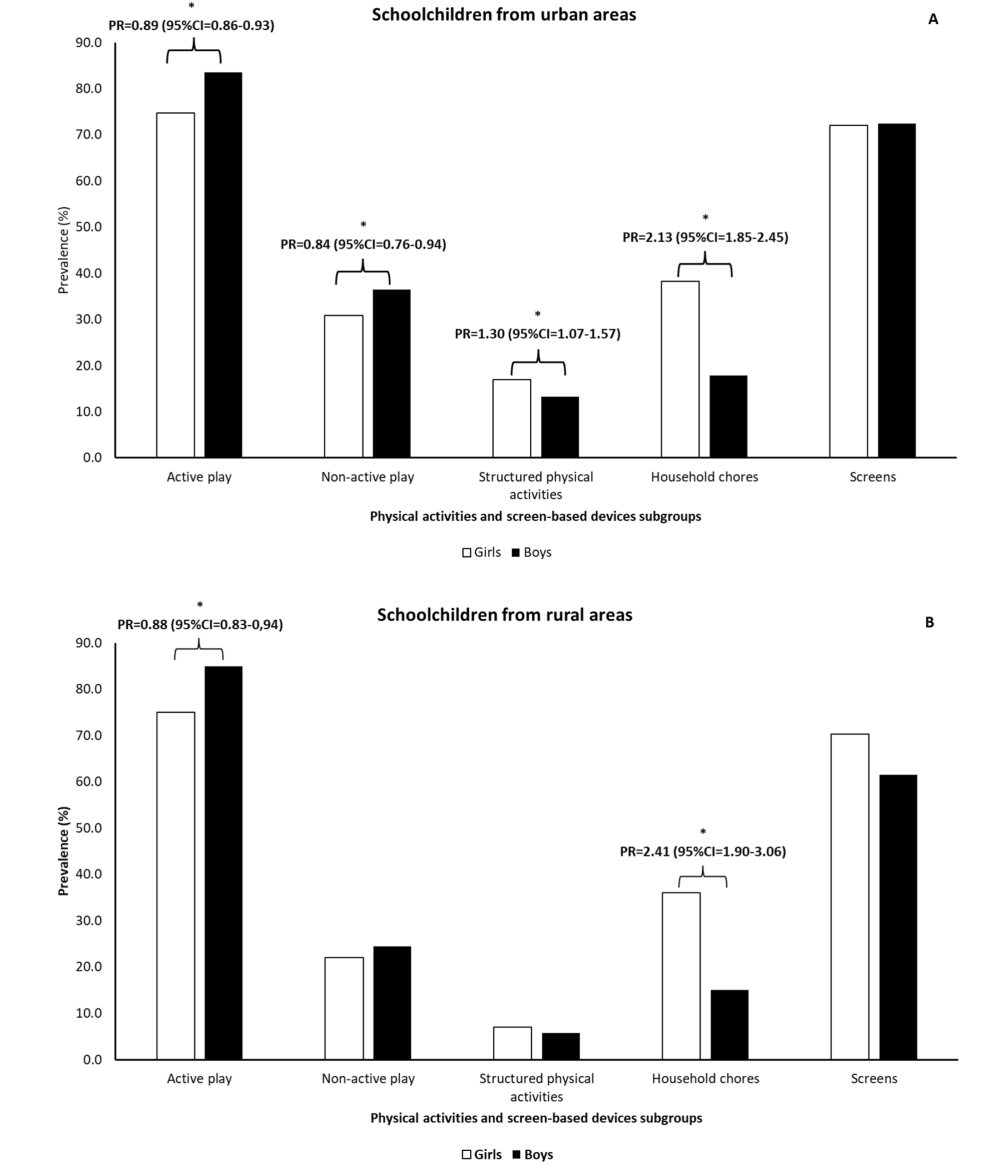
Relative gender inequalities in physical activities and use of screen-based devices subgroups among schoolchildren from urban (A) and rural areas (B). Feira de Santana, Bahia, Brazil

When evaluating subgroups of physical activities and screen usage, the prevalence of *household chores* among girls was more than twice as high as among boys while active play was 12% less reported when compared to boys. There was no statistically significant difference in the self-reports of *nonactive play*, *structured physical activities*, and *screens* (Fig. 3). No interactions were observed between age, overweight status, and gender.

## Discussion

In the current study, we analyzed gender inequalities in types of physical activities and in the use of screen-based devices among schoolchildren from public schools in urban and rural areas, in a large city in the Northeast region of Brazil. Our findings presented a lower prevalence of *active play* among girls. Moreover, their participation in *household chores* was double that observed among boys, both in urban and rural areas.

By and large, in urban areas, girls also participated less in *nonactive play*, but exhibited a higher prevalence of *structured physical activities*, especially ballet– a trend that was particularly noticeable among those in the age group of 7–9 years. Girls in urban and rural areas self-reported more exposure to TV and less exposure to video games. However, there was no gender inequality in the prevalence of daily use of *screens* among schoolchildren in urban and rural areas, i.e., the daily sum of all reports of the screen-based devices included (TV, cell phone, computer, and video game).

Gender social norms influence the participation of girls and boys in physical activity and sports [29] likewise they also reinforce the notion that household chores are exclusively performed by girls. UNICEF data show that around the world, girls between the ages of five and 14 spend 40% more of their daily time on household activities than to boys. This inequality varies between countries but seems to be more pronounced in less developed ones [30].

The greater responsibility for performing tasks such as sweeping, washing dishes, fetching water and firewood, or even taking care of younger siblings during childhood and adolescence is aligned with the social role attributed to women in adulthood and can even influence employment statistics, since in Brazil women represent 92% of the people employed in domestic work [31]. Moreover, investing more hours per day in household chores reduces the opportunities for girls to engage in physical activities and sports [32] with a potential effect on screen exposure, since these activities are performed in the most confined space of the home.

It is important to highlight that our results may have been affected by the effects of the COVID-19 pandemic period on children’s movement behaviors since the data referring to the sample of rural areas were produced in the post-pandemic period.

In a study analyzing changes in the physical activity patterns of 10-to-11-year-olds in the U.S. as a result of the pandemic, Walker et al. (2023) [33] showed that frequent use of electronic devices at home has become a common habit, especially among girls and children of lower socioeconomic status, whose families tend to favor a sedentary lifestyle at home instead of physical activity, giving children higher access to television and less access to portable toys, such as bicycles [33].

This result may reveal potential differences in behavior patterns due to the COVID-19 pandemic. However, our data from the sample of rural areas (post-pandemic period), revealed a pattern of gender inequalities in the types of physical activity and use of screen-based devices similar to those observed among children in urban areas in the pre-pandemic period. This suggests that the patterns observed in the sample of rural areas preexisted with the COVID-19 pandemic and persisted despite it.

We also observed higher participation of girls in structured physical activities in urban areas, especially ballet among girls aged 7–9 years. This result may reflect the higher availability of ballet schools and ballet teaching projects in schools in urban areas, compared to rural areas. Additionally, in rural areas, long distances traveled to sports facilities, lack of public transportation, costs related to physical activities and transportation, and lower family income can be barriers for girls to participate in structured physical activities [34, 35].

Our results also showed lower participation of girls in active play, a grouping that included sports and activities that require skills for handling balls and equipment such as bicycles and skates/skateboards/scooters and are performed in the presence of other children.

Previous studies show that the lower participation of girls in physical activities is due to intrapersonal factors, such as low self-confidence in their physical abilities, discomfort with the fact that their bodies are being observed during practice, and fear of being compared with more skilled subjects [36]. On the other hand, from childhood, girls are more stimulated to perform more sedentary activities and to be reclusive at home and are less stimulated to participate in games that involve sports practices and physical vigor compared to boys [37].

Overexposure to electronic screen devices has become a common behavior among children and teens around the world and has been accentuated since the COVID-19 pandemic. The main screen-based devices most used by children and adolescents from six to 14 years of age include TV, computers, and cell phones/tablets [38]. In our study, TV and cell phones were the most reported devices. Gender differences in TV use are still not consistent in the literature, but girls in our samples exhibited a higher percentage of TV use than boys, both in urban and rural areas. On the other hand, the use of computers and video games was less frequent in the samples and higher among boys.

Limitations of current study includes the use of a subjective measure of physical activity and daily use of screens. To measure these behaviors, we used a self-report instrument, which may be subject to potential memory and social desirability biases. In addition, factors such as the effect of crime, safety, and distance for schools/physical activity facilities were not investigated. These factors can moderate or mediate the associations evaluated.

Regular attendance in classes was inclusion criteria in both surveys and this approach may have introduced a potential bias in our study. Students from disadvantaged backgrounds are less likely to attend classes regularly and this can be more frequent to belong to a particular gender. To mitigate this potential bias, we extended the data collection period in each participating school by up to an additional week to allow students who missed classes on previous data collection days to also participate.

The strengths of the study include: (1) the description of gender inequalities in specific types of physical activity and use of screen-based devices; (2) the use of representative samples of the population of schoolchildren from urban and rural areas; and (3) the presentation of the results in terms of absolute and relative inequalities.

Presenting gender inequalities in the specific types of physical activity and in the use of screen-based devices is an approach that provides detailed and complementary information to the evaluation of inequalities in the level of physical activity of children and adolescents. In conjunction with evidence from research using accelerometry, such as a recent study published by Kretschmer et al., (2023) [39], our findings may help to more clearly understand the inequality in physical activity between girls and boys, which can be driven by moderate to vigorous physical activity, including participation in active play and sports, which was lower among the girls in our study.

Strategies and policies to promote physical activity in the population– like the Brazilian Health Academy Program [40] and School Health Program [41]– can benefit from knowledge of the preferences and contexts of physical activity, which can reflect cultural influences and inequalities in access to facilities and equipment^11^. These programs should focus on the aims of the Global Action Plan on Physical Activity 2018–2030 [42], in the expanding the construction and requalification of sports facilities in schools and near homes, enhancing physical education and school-based programs, and implementing communitywide initiatives, with prioritizing and valorizing girls’ participation.

Furthermore, the findings of the current study can be generalized and used in the formulation of local strategies to promote the participation of girls in several types of physical activities and sports, both in urban and rural areas. To the best of our knowledge, this is the first Brazilian study to present inequalities in different types of physical activities between schoolchildren from urban and rural areas.

Rural areas in Brazil have disadvantages in terms of infrastructure and availability of resources, services and basic inputs when compared to urban areas [43] and this unfavorable condition reflects the lower availability of sports facilities in rural areas and can impact engagement in different types of physical activity and sports.

Another strength of the study was the presentation of inequalities in absolute and relative terms, which provide valuable information for future studies of time trends. In this approach, important fluctuations in inequalities can be noted in absolute values, even if they are not captured in relative terms [44].

Our future research priorities must include an evaluation of the effect of the built environment at schools and near homes on gender inequalities in physical activity among Brazilian schoolchildren, including an evaluation effect of distance to sports facilities, access to means of transport, and crime.

## Conclusion

The findings of the study made it possible to evidence important gender inequalities in the types of physical activities practiced by students from urban and rural public schools. Girls from urban and rural areas exhibited lower participation in *active play*, but their participation in *household chores* was double that observed among boys. Moreover, in urban areas, girls also participated less in *nonactive play*, but exhibited a higher prevalence of *structured physical activity*.

The prevalence of screen-based device use was not different between girls and boys from urban and rural areas. Inequalities in the practice of different types of physical activity may be due to social gender norms, restriction of girls’ participation in sports, such as soccer, and should be considered as potential determinants of the differences noted in the level of physical activity of girls and boys.

### Electronic supplementary material

Below is the link to the electronic supplementary material.

Supplementary Material 1

Supplementary Material 2

## Data Availability

The datasets generated and/or analyzed during the current study are not publicly available due to miss authorization of the Research Ethics Council but are available from the corresponding author upon reasonable request.

## Abbreviations

95% CI: 95% confidence intervals
BMI: Body Mass Index
CAAE: *Certificado de Apresentação de Apreciação Ética* (Certificate of Presentation of Ethical Review)
*CAPES*: *Coordenação de Aperfeiçoamento de Pessoal de Nível Superior* (Higher Education Personnel Improvement Coordination)
*CNS*: *Conselho Nacional de Saúde* (National Health Council)
Diff.: Difference
*IBGE*: *Instituto Brasileiro de Geografia e Estatística* (Brazilian Institute of Geography and Statistics)
IOTF: International Obesity Task Force
p.p.: Percentage points
PA: Physical activity
*PeNSE*: *Pesquisa Nacional de Saúde do Escolar* (National School Health Survey)
PR: Prevalence Ratios
SD: Standard Deviation
UNICEF: United Nations Children’s Fund
VIGITEL Brasil: *Vigilância de Fatores de Risco e Proteção para Doenças Crônicas por Inquérito Telefônico* (Brazilian System for Surveillance of Risk and Protective Factors for Chronic Diseases via Telephone Survey)
Web-CAAFE: Food Intake and Physical Activity of School Children
