# Supplementary material for: Do girls wash dishes and boys play sports? Gender inequalities in physical activity and in the use of screen-based devices among schoolchildren from urban and rural areas in Brazil
Source: BMC Public Health. 2024 Jan 16;24:196. doi: 10.1186/s12889-024-17672-1 (PMC10792968; doi:10.1186/s12889-024-17672-1)
Supplement: Supplementary file 1 — Supplementary Material 1 [file 12889_2024_17672_MOESM1_ESM.docx]

**Table 1S**. Gender inequalities in physical activities and use of screen-based devices among schoolchildren from urban areas. Feira de Santana, Bahia, Brazil.

| **Physical activities and screen-based devices** | **Schoolchildren from Urban areas (n=2,479)** | | | | | | |
| --- | --- | --- | --- | --- | --- | --- | --- |
|  | **Girls** | **95% CI** | **Boys** | **95% CI** | **Diff.** | **PR (95% CI)** | **p value** |
| TV | 50.5 | 47.8-53.2 | 42.3 | 39.5-45.2 | 8.2 | 1.19 (1.10-1.30) | 0.000 |
| Cell phone | 49.4 | 46.7-52.1 | 48.6 | 45.8-51.5 | 0.8 | 1.01 (0.94-1.10) | 0.750 |
| Computer | 9.4 | 7.9-11.1 | 13.8 | 11.9-15.9 | -4.4 | 0.69 (0.55-0.85) | 0.001 |
| Video game | 2.7 | 2.0-3.8 | 17.7 | 15.6-20.0 | -15.0 | 0.15 (0.11-0.22) | 0.000 |
| Playing marbles | 10.2 | 8.6-11.9 | 7.5 | 6.1-9.2 | 2.7 | 1.35 (1.04-1.74) | 0.023 |
| Board games | 3.0 | 2.2-4.0 | 5.2 | 4.0-6.6 | -2.2 | 0.57 (0.38-0.84) | 0.005 |
| Playing with dolls/action figures | 17.6 | 15.6-19.7 | 6.9 | 5.6-8.5 | 10.7 | 2.54 (2.00-3.23) | 0.000 |
| Playing with toy cars | 0.6 | 0.3-1.2 | 9.0 | 7.5-10.8 | -8.4 | 0.07 (0.03-0.14) | 0.000 |
| Spinning top/bayblade | 1.7 | 1.1-2.5 | 16.7 | 14.7-19.0 | -15.0 | 0.10 (0.06-0.15) | 0.000 |
| Listening to music | 9.9 | 8.4-11.7 | 9.7 | 8.1-11.5 | 0.2 | 1.03 (0.81-1.31) | 0.808 |
| Playing musical instrument | 3.8 | 2.9-5.0 | 2.3 | 1.6-3.4 | 1.5 | 1.62 (1.02-2.57) | 0.042 |
| Play catch-up | 32.1 | 29.6-34.6 | 20.8 | 18.5-23.2 | 11.3 | 1.54 (1.34-1.77) | 0.000 |
| Dancing | 11.9 | 10.3-13.8 | 3.4 | 2.5-4.7 | 8.5 | 3.46 (2.46-4.85) | 0.000 |
| Hopscotch | 5.7 | 4.6-7.1 | 12.3 | 10.5-14.4 | -6.6 | 0.45 (0.35-0.59) | 0.000 |
| Gymnastics | 9.2 | 7.8-10.9 | 5.4 | 4.3-6.9 | 3.8 | 1.71 (1.27-2.30) | 0.000 |
| Elastics | 6.5 | 5.3-8.0 | 1.5 | 1.0-2.4 | 5.0 | 4.18 (2.53-6.91) | 0.000 |
| Playing in the park | 8.9 | 7.4-10.5 | 6.4 | 5.1-7.9 | 2.5 | 1.40 (1.06-1.85) | 0.019 |
| Playing in the water/Swimming | 8.2 | 6.8-9.8 | 7.6 | 6.2-9.3 | 0.6 | 1.08 (0.83-1.42) | 0.560 |
| Rollerblading/Skateboarding/Riding a scooter | 6.1 | 5.0-7.6 | 6.3 | 5.0-7.8 | -0.2 | 0.98 (0.72-1.33) | 0.908 |
| Flying a kite | 0.8 | 0.4-1.4 | 6.3 | 5.0-7.8 | -5.5 | 0.12 (0.06-0.23) | 0.000 |
| Dodgeball | 3.0 | 2.2-4.0 | 5.0 | 3.9-6.4 | -2.0 | 0.58 (0.39-0.87) | 0.001 |
| Hide and seek | 15.3 | 13.5-17.4 | 11.6 | 9.9-13.6 | 3.7 | 1.32 (1.07-1.62) | 0.008 |
| Playing with a dog | 8.4 | 7.0-10.0 | 8.3 | 6.8-10.0 | 0.1 | 1.02 (0.79-1.33) | 0.852 |
| Sweeping | 28.9 | 26.5-31.4 | 11.6 | 9.8-13.5 | 17.3 | 2.50 (2.09-2.99) | 0.000 |
| Washing dishes | 28.2 | 25.8-30.7 | 12.9 | 11.1-15.0 | 15.3 | 2.17 (1.83-2.58) | 0.000 |
| Playing with a ball | 31.8 | 23.6-42.8 | 7.1 | 5.7-8.7 | 24.7 | 0.44 (0.31-0.64) | 0.000 |
| Soccer | 6.7 | 5.4-8.1 | 53.3 | 50.4-56.1 | -46.6 | 0.12 (0.10-0.15) | 0.000 |
| Ballet | 14.1 | 12.3-16.1 | 0.4 | 0.2-1.0 | 13.7 | 32.6 (13.5-79.1) | 0.000 |
| Fighting sports | 3.9 | 2.9-5.0 | 12.6 | 10.8-14.6 | -8.7 | 0.31 (0.22-0.42) | 0.000 |
| Jumping rope | 19.5 | 17.4-21.7 | 5.7 | 4.5-7.2 | 13.8 | 3.43 (2.65-4.45) | 0.000 |
| Riding a bicycle | 7.9 | 6.5-9.5 | 13.6 | 11.8-15.7 | -5.7 | 0.58 (0.46-0.73) | 0.000 |

PR: Prevalence Ratio. 95% CI: 95% confidence interval. Diff.: Absolute difference in percentage points between girls' and boys' physical activities and use of screen-based devices prevalence.
