# Supplementary material for: Do girls wash dishes and boys play sports? Gender inequalities in physical activity and in the use of screen-based devices among schoolchildren from urban and rural areas in Brazil
Source: BMC Public Health. 2024 Jan 16;24:196. doi: 10.1186/s12889-024-17672-1 (PMC10792968; doi:10.1186/s12889-024-17672-1)
Supplement: Supplementary file 2 — Supplementary Material 2 [file 12889_2024_17672_MOESM2_ESM.docx]

**Table 2S.** Gender inequalities in physical activities and use of screen-based devices among schoolchildren from rural areas. Feira de Santana, Bahia, Brazil.

| **Physical activities and screen-based devices** | **Schoolchildren from rural areas (n=979)** | | | | | | |
| --- | --- | --- | --- | --- | --- | --- | --- |
|  | **Girls** | **95% CI** | **Boys** | **95% CI** | **Dif.** | **PR (95% CI)** | **p value** |
| TV | 47.5 | 43.1-52.0 | 34.6 | 30.5-39.0 | 12.9 | 1.38 (1.18-1.60) | 0.000 |
| Cell phone | 46.9 | 42.5-51.4 | 41.7 | 37.4-46.2 | 5.2 | 1.13 (0.98-1.30) | 0.085 |
| Computer | 4.5 | 3.0-6.8 | 7.3 | 5.3-10.0 | -2.8 | 0.62 (0.37-1.04) | 0.067 |
| Video game | 2.1 | 1.1-3.8 | 7.7 | 5.7-10.5 | -5.6 | 0.26 (0.13-0.52) | 0.000 |
| Playing marbles | 2.9 | 1.7-4.8 | 15.3 | 12.4-18.8 | -12.4 | 0.19 (0.11-0.33) | 0.000 |
| Board games | 1.4 | 0.7-3.0 | 3.3 | 2.0-5.3 | -1.9 | 0.45 (0.19-1.07) | 0.071 |
| Playing with dolls/action figures | 13.7 | 10.9-17.1 | 2.6 | 1.5-4.5 | 11.1 | 5.12 (2.86-9.14) | 0.000 |
| Playing with toy cars | 0.6 | 0.2-1.9 | 9.4 | 7.1-12.3 | -8.8 | 0.06 (0.02-0.21) | 0.000 |
| Spinning top/bayblade | 0.2 | 0.01-1.4 | 10.2 | 7.8-13.2 | -10.0 | 0.02 (0.003-0.14) | 0.000 |
| Listening to music | 5.9 | 4.2-8.4 | 4.1 | 2.6-6.2 | 1.8 | 1.47 (0.84-2.58) | 0.175 |
| Playing musical instrument | 2.1 | 1.1-3.8 | 1.4 | 0.7-3.0 | 0.7 | 1.45 (0.56-3.76) | 0.447 |
| Play catch-up | 31.4 | 27.4-35.6 | 20.6 | 17.2-24.4 | 10.8 | 1.51 (1.21-1.88) | 0.000 |
| Dancing | 7.6 | 5.5-10.3 | 1.2 | 0.6-2.7 | 6.4 | 6.20 (2.64-14.54) | 0.000 |
| Hopscotch | 14.8 | 11.9-18.2 | 3.9 | 2.5-6.0 | 10.9 | 3.79 (2.32-6.18) | 0.000 |
| Gymnastics | 7.0 | 5.0-9.6 | 2.8 | 1.7-4.8 | 4.2 | 2.46 (1.34-4.54) | 0.004 |
| Elastics | 3.3 | 2.0-5.3 | 1.2 | 0.6-2.7 | 2.1 | 2.68 (1.06-6.80) | 0.038 |
| Playing in the park | 3.7 | 2.3-5.8 | 4.5 | 3.0-6.7 | -0.8 | 0.82 (0.45-1.51) | 0.530 |
| Playing in the water/Swimming | 2.5 | 1.4-4.3 | 4.9 | 3.3-7.2 | -2.4 | 0.50 (0.25-0.99) | 0.048 |
| Rollerblading/Skateboarding/Riding a scooter | 1.4 | 0.7-3.0 | 2.0 | 1.1-3.8 | -0.6 | 0.70 (0.27-1.84) | 0.473 |
| Flying a kite | 2.2 | 1.2-4.0 | 15.9 | 12.9-19.4 | -13.7 | 0.14 (0.08-0.26) | 0.000 |
| Dodgeball | 4.1 | 2.6-6.3 | 2.2 | 1.2-4.0 | 1.9 | 1.84 (0.89-3.80) | 0.102 |
| Hide and seek | 12.9 | 10.2-16.2 | 10.6 | 8.2-13.6 | 2.3 | 1.20 (0.85-1.70) | 0.290 |
| Playing with a dog | 8.8 | 6.6-11.7 | 9.2 | 6.9-12.1 | -0.4 | 0.96 (0.64-1.43) | 0.843 |
| Sweeping | 27.2 | 23.5-31.4 | 9.8 | 7.4-12.8 | 17.4 | 2.80 (2.07-3.80) | 0.000 |
| Washing dishes | 29.9 | 26.0-34.2 | 12.0 | 9.4-15.2 | 17.9 | 2.50 (1.90-3.29) | 0.000 |
| Playing with a ball | 2.5 | 1.4-4.3 | 5.7 | 4.0-8.2 | -3.2 | 0.43 (0.22-0.84) | 0.013 |
| Soccer | 8.6 | 6.4-11.4 | 51.9 | 47.5-56.3 | -43.3 | 0.17-0.12-0.22) | 0.000 |
| Ballet | 4.7 | 3.2-7.0 | 0.2 | 0.016-1.4 | 4.5 | 23.1 (3.12-170.5) | 0.002 |
| Fighting sports | 2.2 | 1.2-4.0 | 5.5 | 3.8-7.9 | -3.3 | 0.40 (0.20-0.81) | 0.010 |
| Jumping rope | 13.1 | 10.4-16.4 | 4.7 | 3.1-7.0 | 8.4 | 2.80 (1.77-4.45) | 0.000 |
| Riding a bicycle | 10.4 | 8.0-13.5 | 14.7 | 11.8-18.1 | -4.3 | 0.71 (0.51-1.00) | 0.050 |

PR: Prevalence Ratio. 95% CI: 95% confidence interval. Diff.: Absolute difference in percentage points between girls' and boys' physical activities and use of screen-based devices prevalence.
